# Prevalence and Antimicrobial Resistance Patterns of *Escherichia coli* Isolated From Broiler Chickens in Sylhet District of Bangladesh

**DOI:** 10.1002/vms3.70576

**Published:** 2025-09-08

**Authors:** Manna Roy, Obaidul Islam, Md Altafur Rahman, Sharmin Sultana Misty, Raju Kurmi, Md Ashraful Islam, Ahsan Raquib, Jahid Hasan Tipu, Md. Anwar Hossain, Md. Siddiqul Islam

**Affiliations:** ^1^ Department of Pharmacology and Toxicology Faculty of Veterinary Animal and Biomedical Sciences, Sylhet Agricultural University Sylhet Bangladesh; ^2^ College of Veterinary Medicine Chungbuk National University Cheongju Chungbuk South Korea; ^3^ Graduate School of Innovation and Practice for Smart Society Hiroshima University Hiroshima Japan; ^4^ Department of Microbiology and Hygiene Faculty of Veterinary Science Bangladesh Agricultural University Mymensingh Bangladesh; ^5^ Faculty of Veterinary Animal and Biomedical Sciences Sylhet Agricultural University Sylhet Bangladesh; ^6^ Laboratory of Veterinary Laboratory Medicine College of Veterinary Medicine Chungbuk National University Cheongju Chungbuk South Korea; ^7^ Department of Health Management Atlantic Veterinary College University of Prince Edward Island Charlottetown Canada; ^8^ Department of Clinical Science Faculty of Medicine University of Bergen Bergen Norway

**Keywords:** Antimicrobial resistance (AMR), Bangladesh, correlation, *Escherichia coli* (*E. coli*), poultry, Sylhet

## Abstract

The emergence of antimicrobial resistance (AMR) *Escherichia coli* in poultry farming is a growing global public health concern, particularly in Bangladesh, where the use of antibiotics remains largely unregulated. This study aimed to determine the prevalence and AMR patterns of *E. coli* isolated from broiler chickens in Sylhet district of Bangladesh and to investigate the network of coexisting resistance traits among the isolates. A total of 130 samples (44 cloacal, 46 faecal, 21 liver and 19 intestinal) were collected from live and dead broiler chickens in the Sylhet district of Bangladesh from July 2020 to June 2021. *E. coli* was detected in 77.7% of samples by standard cultural and biochemical tests, with 65.4% confirmed by polymerase chain reaction (PCR) targeting the *malB* gene. Antibiotic susceptibility testing revealed complete (100%) resistance to tetracycline (TE), cloxacillin (CLOX) and co‐trimoxazole (COT), with 91.8% resistance to erythromycin (E). Gentamicin (GEN) exhibited intermediate resistance (69.4%), whereas azithromycin (AZM) was the most sensitive, with 58.8% of the isolates demonstrating susceptibility. Faecal samples had the highest *E. coli* prevalence (84.8%), and liver samples had the lowest (66.7%). All isolates demonstrated multidrug resistance (MDR) in different combinations, with over one‐third exhibiting resistance to six or more antibiotics. The coexistence network revealed that resistance to TE, CLOX and COT frequently occurred together, whereas GEN exhibited a distinct resistance pattern characterized by limited co‐resistance with other antibiotics. The findings of this study extend beyond local concerns, carrying global implications for food safety, and emphasize the urgent need for stricter antibiotic regulations to mitigate the zoonotic transmission of MDR *E. coli* to humans.

**Summary:**

- The study reported a 77.7% prevalence of *Escherichia coli* in broiler chickens in Sylhet, Bangladesh with alarming resistance patterns, including complete (100%) resistance to several antibiotics (tetracycline, cloxacillin and co‐trimoxazole), underscoring an urgent public health concern.
- The results revealed critical resistance trends, showing that several antibiotics are losing their effectiveness, which could threaten sustainable poultry farming and food safety.
- The correlation and coexistence network analysis identified frequent resistance linkages among specific antibiotics, suggesting shared pathways that could drive co‐selection in resistant *E. coli* populations.
- The study emphasizes the pressing need for stricter antibiotic regulations, enhanced AMR surveillance and improved biosecurity measures to mitigate the spread of multidrug‐resistant *E. coli*, with implications for both human and animal health.

## Graphical abstract

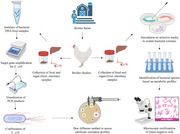

**Abbreviations**
AMC: amoxicillin/clavulanic acid
AMR: antimicrobial resistance
AZM: azithromycin
CIP: ciprofloxacin
CLOX: cloxacillin
COT: co‐trimoxazole
E: erythromycin
GEN: gentamicin
MDR: multidrug‐resistant
TE: tetracycline

## 1 Introduction

The poultry industry is one of the most rapidly growing sectors, contributing significantly to economic growth and ensuring food security worldwide. With an estimated annual production of more than 133 million tonnes of poultry meat globally, this sector plays an imperative role by providing accessible and affordable sources of protein (FAO 2024). In developing countries like Bangladesh, this sector contributes to overcoming malnutrition and alleviating poverty by supplying quality meat and eggs at a reasonable prices and providing employment opportunities (Tipu et al. 2021).

Despite its economic importance, this sector faces considerable challenges from different infectious diseases (Ali et al. 2015; Sagor et al. 2022). A wide range of pathogens, including viruses, bacteria and parasites, affect this sector and hinder its sustainable growth. Among these, *Escherichia coli* (*E. coli*) is one of the most common pathogens affecting poultry, which is a gram‐negative bacterium in the Enterobacteriaceae family (Islam et al. 2023). Most of the strains of *E. coli* are non‐pathogenic and commonly found in the environment and in the normal gut flora of birds (Ramos et al. 2020). However, some pathogenic strains of *E. coli*, such as enteropathogenic (EPEC), enterotoxigenic (ETEC), enteroinvasive (EIEC) and enterohemorrhagic (EHEC), cause illness and are responsible for significant economic losses (Lim et al. 2020). These pathotypes contribute to various disease manifestations—including yolk sac infection, cellulitis, omphalitis, coligranuloma and colibacillosis—that reduce productivity, increase mortality rates and cause substantial economic losses in poultry farming (Islam et al. 2021). Chickens infected with EPEC strains adhere to the intestinal mucosa, causing villus atrophy and malabsorption, leading to diarrhoea and growth retardation in young chicks (Lutful Kabir 2010). Enterotoxins produced by ETEC dysregulate electrolyte balance in the gut microbiota, particularly in young chicks, which leads to episodes of massive, watery diarrhoea and dehydration (Dubreuil et al. 2016). EIEC induces inflammation within the intestinal epithelial cells, which may result in enteritis and reduced performance (Mohamed et al. 2022). Although EHEC is recognized as a human pathogen, certain Shiga toxin‐producing *E. coli* strains have been isolated from chickens (Mellata 2013), raising concerns about their potential to enter the human food chain.

In Bangladesh, antibiotics such as colistin, enrofloxacin, ciprofloxacin, streptomycin, gentamicin, erythromycin, tetracycline, levofloxacin and trimethoprim–sulphamethoxazole are commonly used to treat this pathogen (Al Azad et al. 2019). Additionally, antibiotics are also used in sub‐therapeutic doses by adding them to feed and water for prophylaxis, as a growth promotor and as a risk‐management strategy (Begum et al. 2014). Unfortunately, the use of antibiotics is largely unregulated in Bangladesh, as antibiotics can be bought without the need for veterinary prescriptions (Davies et al. 2024; Masud et al. 2020; Umair et al. 2023). Consequently, the widespread and indiscriminate use of antibiotics has contributed to the development of different antimicrobial resistance (AMR) *E. coli* strains, which is a major public health concern globally (Founou et al. 2016). This practice seriously threatens both human and animal health by driving the development of multidrug‐resistant (MDR) *E. coli* strains, which can be transmitted from animals to humans through direct contact, environmental contamination and consumption of contaminated meat and eggs (Founou et al. 2016; Saiful Islam et al. 2021; Singh et al. 2025). This presents a growing public health challenge, particularly in countries where veterinary oversight is limited.

Although several studies have examined *E. coli* and their associated AMR in poultry across different regions of Bangladesh (Parvin et al. 2020; Rahman et al. 2020; Islam et al. 2023; Ibrahim et al. 2023), limited data are available from Sylhet district, which is a major poultry production hub in Bangladesh. This knowledge gap restrains the development of region‐specific AMR surveillance and control strategies, making it imperative to understand the prevalence of *E. coli* with its resistance patterns in this setting considering public health impact. Therefore, the present study was conducted to determine the prevalence and AMR patterns of *E. coli* isolated from broiler chickens in the Sylhet district of Bangladesh. Secondly, we investigated the MDR profiles of the isolates, along with the correlation and coexistence networks among different antimicrobial agents.

## 2 Materials and Methods

### 2.1 Study Area and Sample Size

**FIGURE 1:**
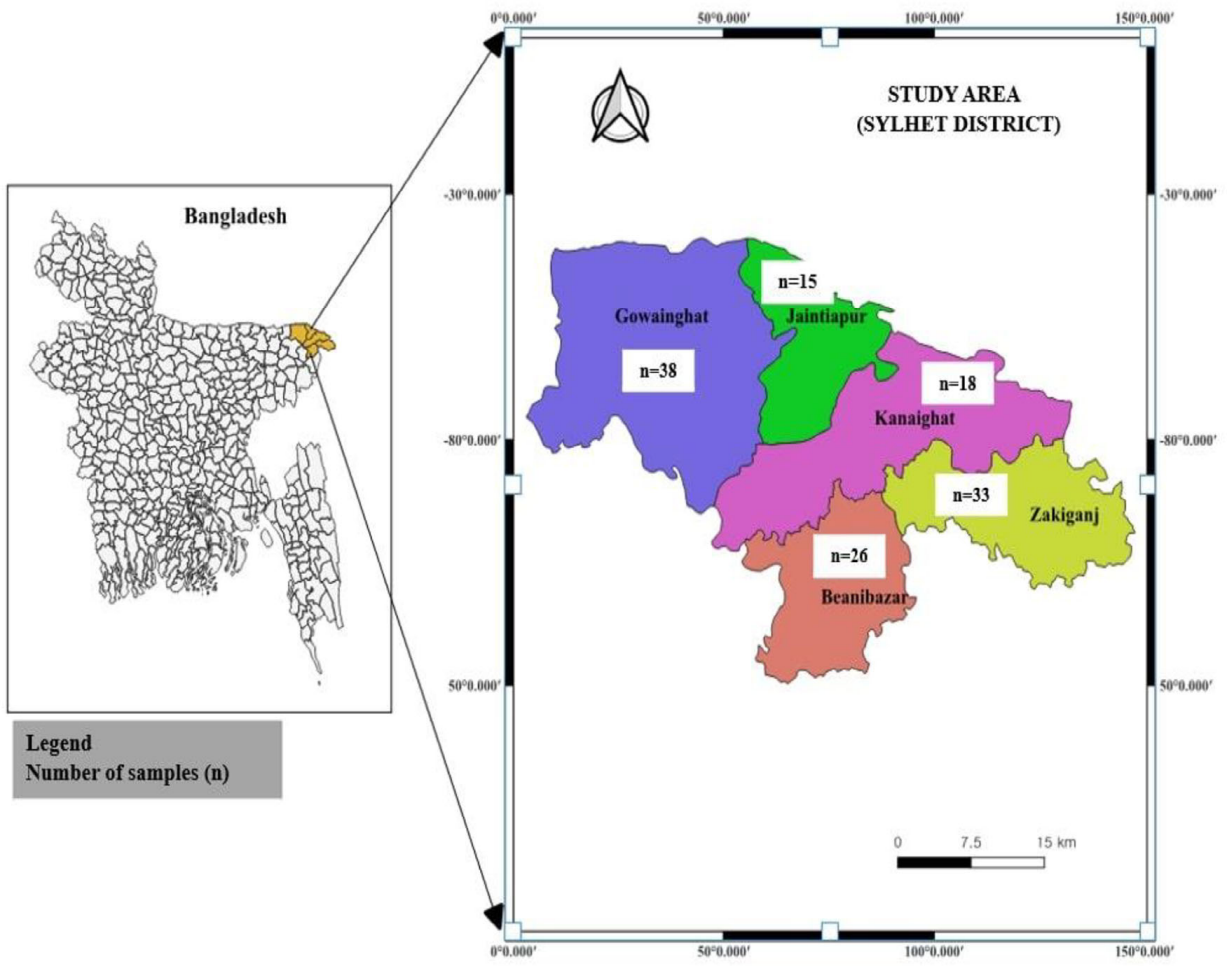
Map indicating the sampling sites across five different farms in the Sylhet district of Bangladesh: Farm A in Beanibazar; Farm B in Zakiganj; Farm C in Gowainghat; Farm D in Jaintapur; and Farm E in Kanaighat.

A total of 130 samples were collected from five different poultry farms (farms A–E) located in the Sylhet district of Bangladesh from July 2020 to June 2021 (Figure 1). The geographical location of these farms is presented in Table S1. Of these, 90 samples were obtained from live birds (44 cloacal samples and 46 freshly dropped faecal samples), whereas 40 samples were collected from dead birds (21 liver samples and 19 intestinal samples) (Table S2).

### 2.2 Sample and Stock Culture Preparation

The collected samples were immediately preserved in an ice box to maintain their integrity and to prevent degradation. The liver and intestinal samples of dead birds were ground to prepare a 10% suspension using phosphate‐buffered saline (PBS). Following that, 1 mL of each sample was transferred to 9 mL of nutrient broth (NB) for the growth of *E. coli*. Additionally, the cloacal swab and faecal samples from live birds were collected using sterile cotton swabs from at least five birds per farm. The samples were pooled and placed into PBS in a test tube. Each type of sample was then transferred to NB for enrichment, assigned a specific tag number and incubated at 37°C for the growth of *E. coli*.

### 2.3 Isolation and Identification of *E. coli*

The isolation of *E. coli* was based on culture on MacConkey agar and eosin–methylene blue (EMB) agar plates. Initially, freshly grown NB cultures were streaked on MacConkey and EMB agar media with a sterile inoculating loop in aseptic conditions and incubated at 37°C for 24 h. Large pink‐coloured colonies on MacConkey agar and single green‐coloured metallic sheen colonies on EMB agar indicated the growth of *E. coli*. For further confirmation, the selected colonies were subjected to Gram's staining for morphological study, and different biochemical tests, namely– indole test, methyl red (MR) test, Voges–Proskauer (VP) test and catalase test, were performed as standard procedure (Ali et al. 2021).

### 2.4 DNA Extraction and Molecular Detection of *E. coli*

DNA was extracted by a simple boiling method with slight modifications (Dashti et al. 2009). Briefly, a single pure colony was inoculated in NB broth culture and incubated at 37°C for 24 h. Afterwards, 1 mL of broth culture was transferred into an Eppendorf tube and centrifuged at 10,000 rpm for 3 min. The supernatant was discarded and resuspended in 1 mL of sterile distilled water (DW). The mixture was vortexed and centrifuged again, and the supernatant was discarded. The pellet was then resuspended in 100 µL DW. The tube was placed in boiling water for 15 min, followed by immediate cooling on ice for 10 min. The tube was vortexed and centrifuged once again at 10,000 rpm for 10 min. The resulting supernatant was collected as template DNA for polymerase chain reaction (PCR) and stored at −20°C until further use.

For confirmation of the presence of *E. coli*, we conducted a conventional PCR targeting the maltose operon protein B (*malB*) gene of *E. coli* utilizing previously published primers, Eco‐1 (5′‐GACCTCGGTTTAGTTCACAGA‐3′) and Eco‐2 (5′‐CACACGCTGACGCTGACCA‐3′) (Wang et al. 1996). To amplify the target region of *E. coli*, the PCR reaction was performed with a total volume of 20 µL consisting of 10 µL of Master Mix 2X (Promega, USA), 1 µL of each of the primers, 3 µL of extracted genomic DNA and 5 µL of nuclease‐free water (NFW). After thoroughly mixing and settling using a mini‐centrifuge, we performed the PCR reaction in a 96‐well thermocycler (Thermo Fisher Scientific, USA) with the following cycling conditions: 94°C for 15 s (initial denaturation), followed by 35 cycles, each consisting of 94°C for 3 s (denaturation), 50°C for 10 s (annealing) and 74°C for 35 s (elongation), with a final elongation at 74°C for 2 min. To confirm the successful amplification of targeted DNA fragments (expected amplicon size 585 bp), we analysed the amplified PCR products using 1.5% agarose gel electrophoresis  stained with ethidium bromide (0.5 µg/mL). Finally, we ran the gel at 90 V for approximately 1.5 h and visualized the DNA amplicons under a UV transilluminator (Biometra, Germany).

### 2.5 Antibiotic Susceptibility Test Using the Kirby–Bauer Disc Diffusion Method

One PCR‐confirmed *E. coli* isolate from each sample was subjected to antibiotic susceptibility testing using the Kirby–Bauer disc diffusion method described by the Clinical and Laboratory Standards Institute (CLSI 2020). The bacterial concentration was initially adjusted to 0.5 McFarland standard and spread on Mueller–Hinton agar medium (HiMedia, Mumbai, India). Isolates were tested against eight commonly used antibiotics from six classes: tetracycline (TE), azithromycin (AZM), erythromycin (E), gentamicin (GEN), co‐trimoxazole (COT), ciprofloxacin (CIP), cloxacillin (CLOX) and amoxicillin/clavulanic acid (AMC) (Table S3). All results were interpreted as susceptible, intermediate and resistant per CLSI guidelines, 2020 (Table S4). Isolates that exhibited resistance to at least three antibiotics were considered as MDR.

### 2.6 Data Analysis

The prevalence of *E. coli* among different groups was compared using the chi‐square test. Additionally, a post hoc chi‐square test was employed to perform pairwise comparisons between different farms and samples. Spearman's rank correlation was used to find out the relation among resistance patterns (sensitive, intermediate and resistant) of different antibiotics. The co‐occurrence of resistance patterns across different antibiotics was presented in a network graph where each node represented antibiotics, and the frequency of co‐occurrence was represented by edges. All statistical analysis and visualization were performed in R software (version 4.2.2), and BioRender was used to create the graphical abstract.

## 3 Results

### 3.1 Isolation and Identification of *E. coli* Using Cultural, Biochemical and PCR Methods

Among the 130 collected samples, 101 (77.7%) samples were positive for *E. coli* by different cultural and biochemical tests exhibiting characteristic features of *E. coli*, and 85 isolates (65.4%) were confirmed positive in PCR (Figure S1).

### 3.2 Prevalence of *E. coli* Infections in Broiler Chickens

**FIGURE 2:**
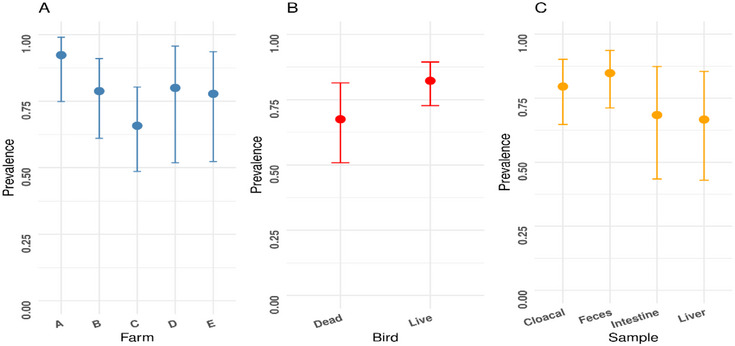
Prevalence of *E. coli* based on: (A) five different farms, (B) status of birds, and (C) sample types in the Sylhet district of Bangladesh during the study period.

The study demonstrated notable variations in prevalence of *E. coli* based on the farms, bird status and sample types (Figure 2). Farm A had the highest prevalence at 92.3%, whereas farm C showed the lowest at 65.8%. Regarding bird status, live birds had a higher prevalence of *E. coli* (82.2%) compared to dead birds (67.5%). Among different sample types, faecal samples exhibited the highest prevalence at 84.8%, whereas the lowest was recorded in liver samples at 66.7%.

### 3.3 AMR Patterns of *E. coli* Isolates

**FIGURE 3:**
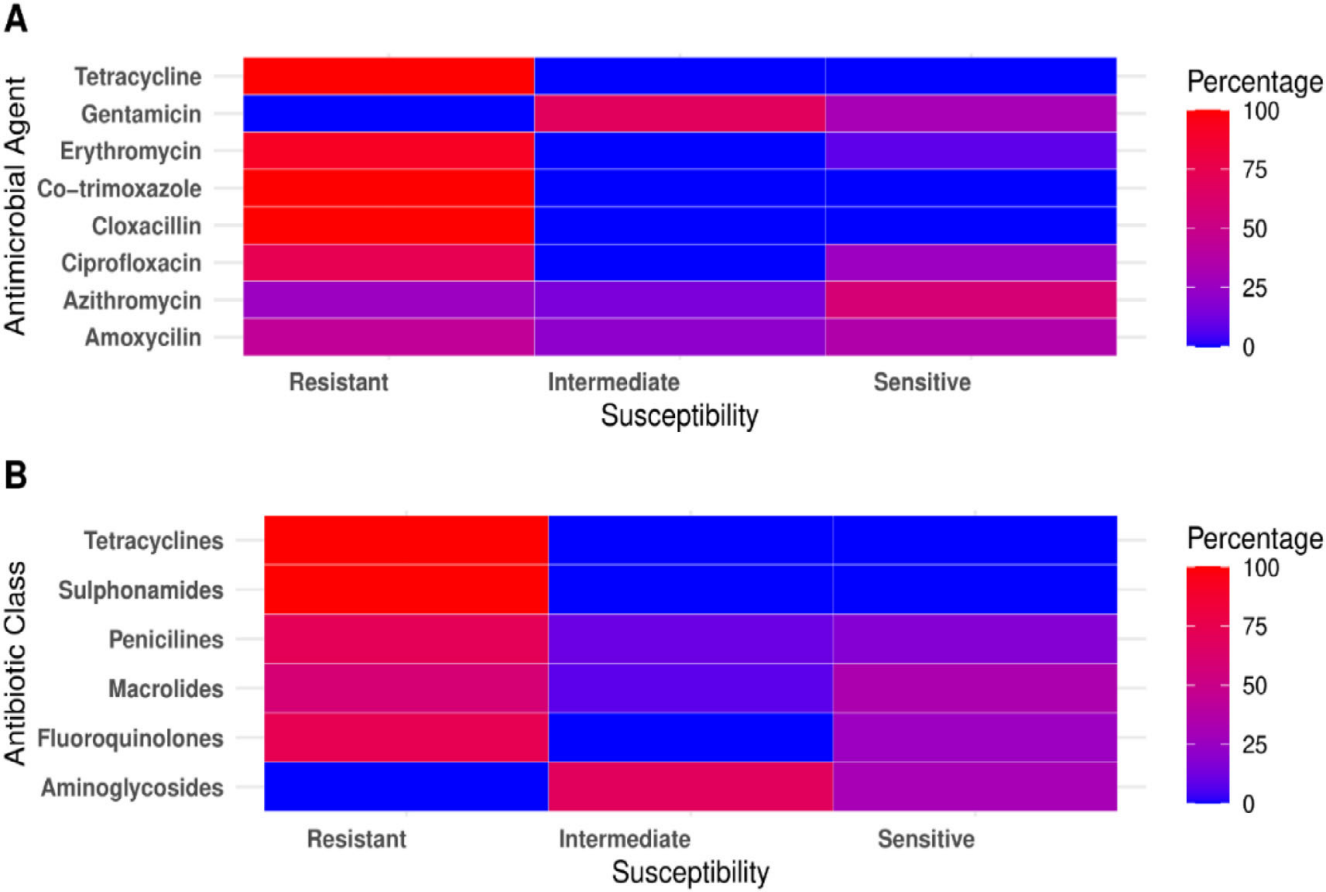
Antimicrobial resistance pattern observed in *E. coli* isolates during the study period, categorized by (A) individual antimicrobial agents, and (B) their corresponding antibiotic class.

In this study, we evaluated the antibiotic susceptibility of eight commonly used antibiotics against 85 isolates of *E. coli* that tested positive in PCR (Figure 3A and Table S5). The findings revealed alarming resistance patterns, as all isolates exhibited 100% resistance to TE, CLOX and COT, raising significant concerns about the effectiveness of these commonly used antibiotics. Similarly, 91.8% of the isolates were resistant to E. In contrast, none of the isolates were resistant to GEN, although 69.4% displayed an intermediate resistance profile. Interestingly, AZM and AMC were more effective, with 58.8% and 35.3% of the isolates classified as sensitive respectively.

The eight antibiotics used in this study were grouped into six different antibiotic classes, and their susceptibility was also thoroughly evaluated (Figure 3B). Tetracyclines and sulphonamides emerged with the highest resistance rates, raising concerns about their efficacy. In contrast, macrolides displayed greater sensitivity, whereas aminoglycosides exhibited intermediate resistance, reflecting a more nuanced pattern.

**FIGURE 4:**
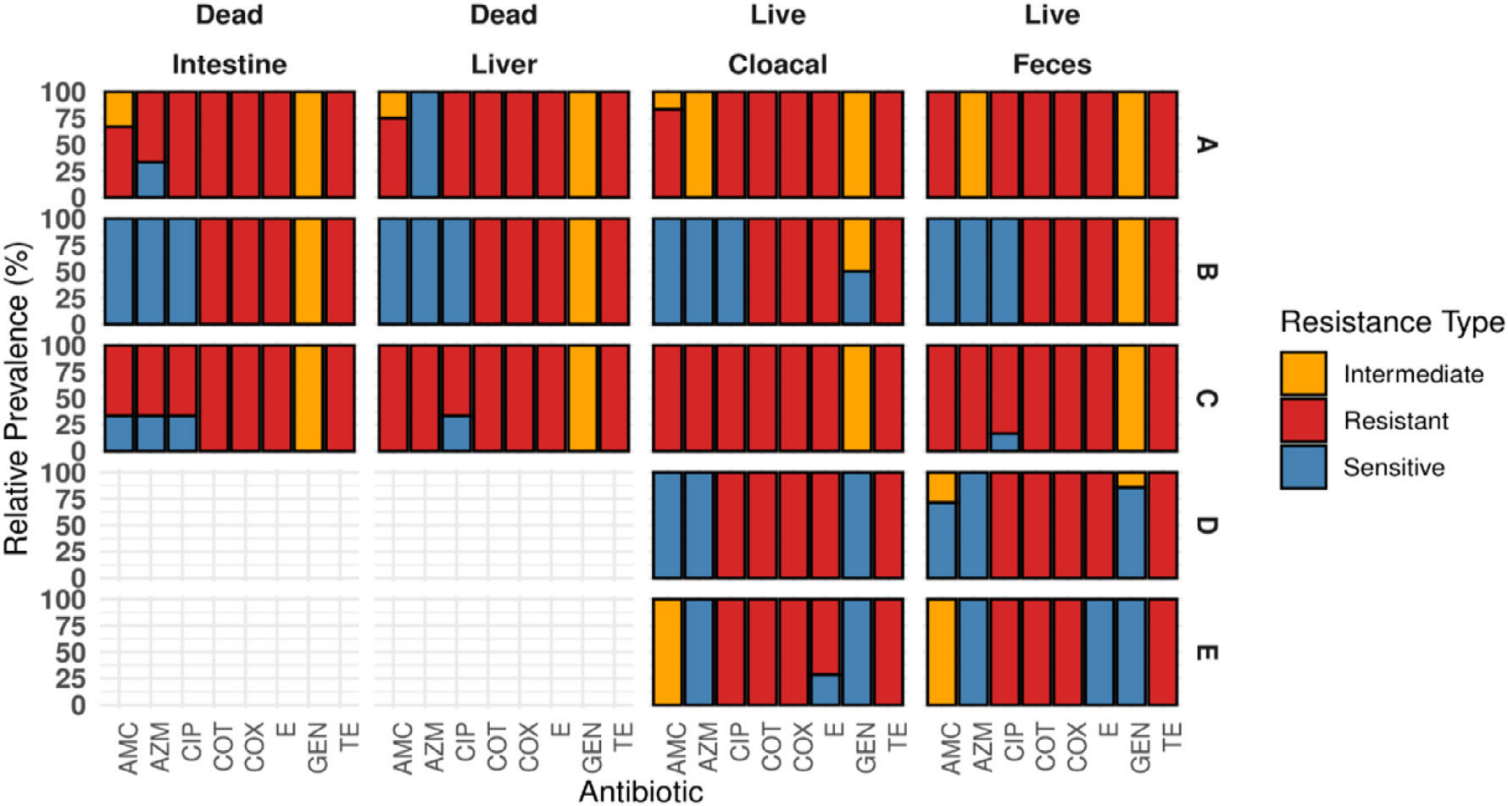
Relative prevalence of antimicrobial resistance pattern of *E. coli* isolates across different farms, bird status and sample types in the Sylhet district of Bangladesh during this study period. AMC, amoxicillin/clavulanic acid; AZM, azithromycin; CIP, ciprofloxacin; COT, co‐trimoxazole; GEN, gentamicin; TE, tetracycline.

**FIGURE 5:**
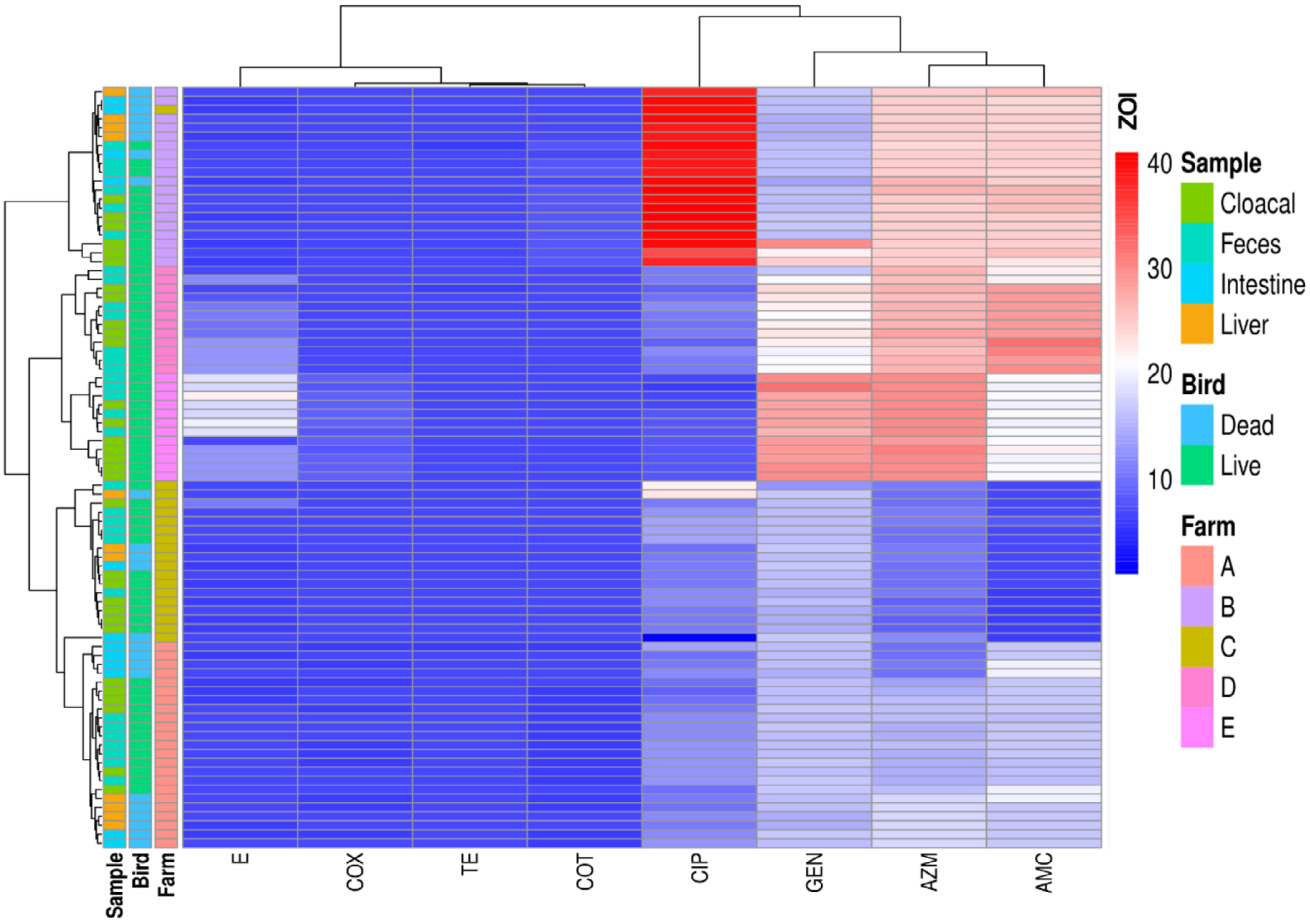
Resistance profile of 85 isolates of *E. coli*, with clustering based on resistance pattern, sample types, the status of bird and different farms of Sylhet district of Bangladesh during the study period. AMC, amoxicillin/clavulanic acid; AZM, azithromycin; CIP, ciprofloxacin; COT, co‐trimoxazole; GEN, gentamicin; TE, tetracycline; ZOI, zone of inhibition.

We analysed the resistance patterns of the used antibiotics across five different farms (A–E), the status of the birds (live/death) and four sample types (intestine, liver, cloacal and faecal) from broiler chickens (Figure 4 and Figure 5). The majority of the isolates across all farms exhibited high resistance to TE, CLOX and COT, regardless of sample type or bird status. Comparatively, farm A demonstrated higher resistance, the isolates of farm C displayed a more uniform pattern of resistance across multiple antibiotics. In contrast, farm B demonstrated more sensitivity across a few antibiotics and sample types compared to other farms. GEN showed variability in resistance profiles across sample types and bird status. Although most of the isolates from dead birds (particularly intestine and liver) exhibited intermediate resistance, a few samples from live birds (particularly cloacal and faecal samples) demonstrated susceptibility. The dead birds showed a higher proportion of resistance across most of the antibiotics compared to the live bird samples. This trend was particularly pronounced in farms A and B.

### 3.4 MDR Patterns of *E. coli* Isolates

All 85 *E. coli* isolates exhibited MDR, with resistance to a minimum of at least four of the antibiotics tested in this study. The distribution and proportion of *E. coli* isolates resistant to specific antibiotic combinations are presented in Table 1. The most common combination included E, TE, CLOX and COT—found in 23.5% of the isolates (*n* = 20). This was succeeded by CIP, E, TE, CLOX and COT resistance in 22.4% of the isolates (*n* = 19). Interestingly, more than one‐third of the isolates were resistant to six or more antibiotics, indicating a high diversity of resistance among the isolates.

**TABLE 1:** Frequency and proportion of multidrug‐resistant (MDR) *E. coli* isolates based on resistance to different antibiotic combinations reported in this study.

Multidrug‐resistant pattern	Number of isolates	Proportion (%)
Ciprofloxacin + erythromycin + tetracycline + amoxycillin/clavulanic + cloxacillin + co‐trimoxazole	17	20
Ciprofloxacin + erythromycin + tetracycline + cloxacillin + co‐trimoxazole	19	22.4
Erythromycin + tetracycline + cloxacillin + co‐trimoxazole	20	23.5
Ciprofloxacin + erythromycin + azithromycin + tetracycline + amoxycillin/clavulanic + cloxacillin + co‐trimoxazole	18	21.2
Ciprofloxacin + tetracycline + cloxacillin + co‐trimoxazole	7	8.2
Erythromycin + azithromycin + tetracycline + amoxycillin/clavulanic + cloxacillin + co‐trimoxazole	2	2.4
Ciprofloxacin + erythromycin + azithromycin + tetracycline + cloxacillin + co‐trimoxazole	2	2.4

### 3.5 The Correlation and Coexistence Network of Antimicrobial Agents

**FIGURE 6:**
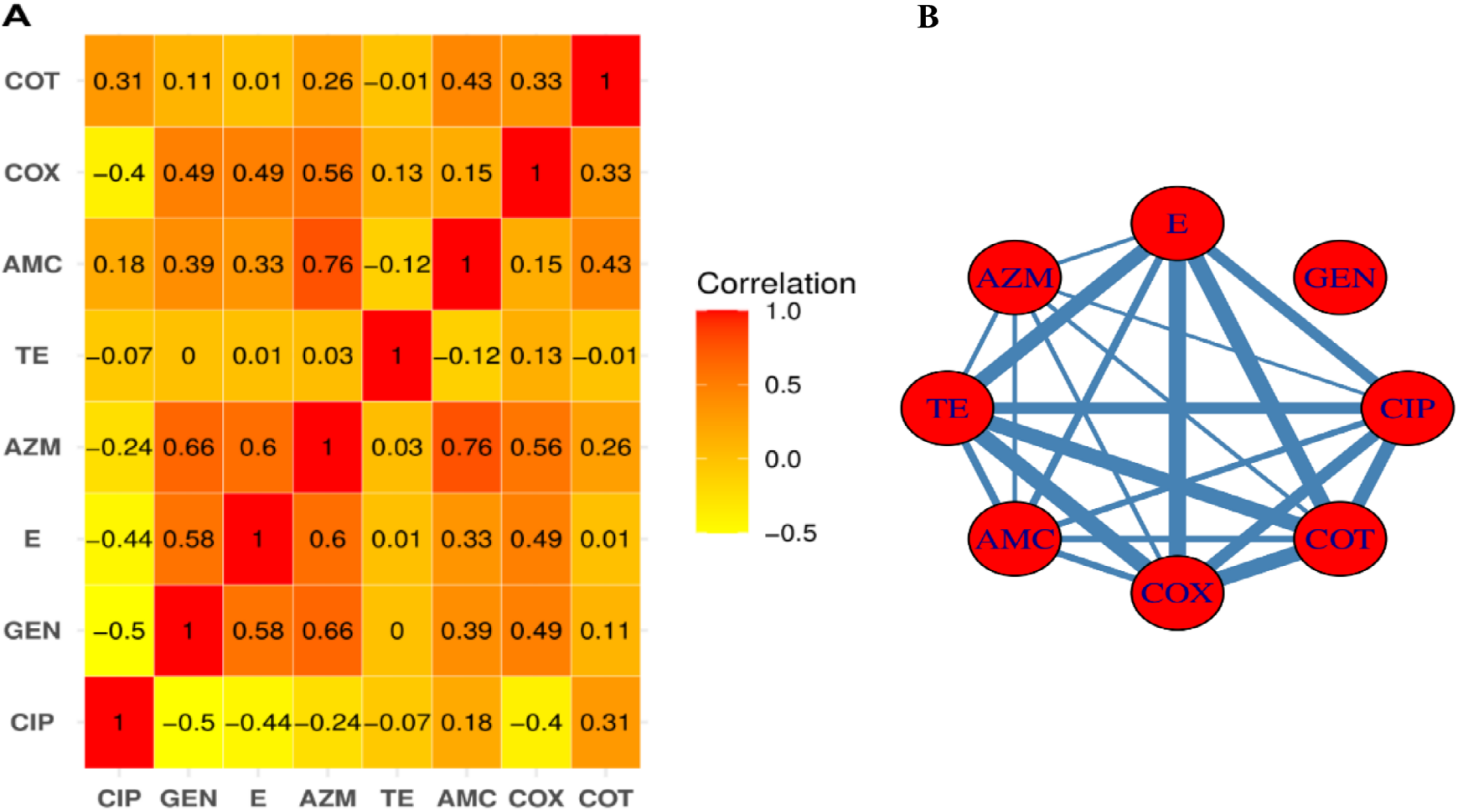
(A) Correlation of antimicrobial agents employed in this study, where −0.5 indicated a negative correlation and 1 indicated a positive correlation. (B) coexistence network of used antimicrobial agents, where the size of the edges represents the frequency of coexistence among the antimicrobial agents. AMC, amoxicillin/clavulanic acid; AZM, azithromycin; CIP, ciprofloxacin; COT, co‐trimoxazole; GEN, gentamicin; TE, tetracycline.

The correlation and coexistence patterns among the tested antimicrobial agents used in this study on *E. coli* are presented in Figure 6. The heatmap (Figure 6A) illustrated notable correlations among the used antibiotics, ranging from −0.5 to 0.76. Notably, a strong positive correlation was observed for AZM with AMC (0.76) and GEN (0.66). Interestingly, AZM and AMC exhibited a positive correlation with nearly all other antibiotics. In contrast, certain antibiotics displayed striking negative correlations, for instance− CIP showed negative correlations with almost all other agents, particularly GEN (−0.5) and E (−0.44).

The coexistence networks (Figure 6B) denoted simultaneous resistance to several antibiotics, with thicker lines indicating stronger associations and thinner lines indicating less common coexistence. The network revealed distinct patterns, highlighting frequent co‐selection among certain antibiotics like TE, CLOX, COT and E, whereas GEN appeared isolated from other agents in the network, indicating different resistance pathways.

## 4 Discussion

The present study provides valuable insights into the prevalence and AMR of *E. coli* in the Sylhet district of Bangladesh isolated from broiler chickens, shedding light on the growing concern regarding MDR bacteria in poultry production.

The isolates exhibited characteristic smooth, circular, greenish‐black colonies with a metallic sheen on EMB agar and pink colonies on MacConkey agar, consistent with standard descriptions (CLSI 1993). Morphologically, the isolates were identified as gram‐negative, small, rod‐shaped bacteria, arranged singly or in pairs, as similarly reported by Tuttle et al. (2021). Biochemical characterization further supported the identification, showing positive results for the catalase, MR and indole tests, and a negative result for the VP test. These findings are consistent with previous studies, further enhancing our study's credibility (Quinn et al. 2011).

The overall prevalence of *E. coli* observed in this study was 77.7%, which aligns with several previous studies conducted in Bangladesh (Davies et al. 2024; Al‐Salauddin et al. 2015; Mahmud et al. 2018; Parvin et al. 2020; Mandal et al. 2022) and Turkey (Telli et al. 2022). In contrast, our results surpass the lower prevalence rates reported in countries like Sri Lanka, the United States, the United Kingdom and even Bangladesh at 66.8%, 67.9%, 36.4%, 63.5% and 56.5%, respectively (Ranasinghe et al. 2022; Lee et al. 2023; Willis et al. 2023; Rahman et al. 2020; Alam et al. 2023). These variations in prevalence could be attributed due to the differences in environmental conditions, farming practices and antimicrobial usage patterns across geographical locations. The dynamic nature of *E. coli* prevalence highlights the complex interplay between local farming conditions and global food production practices.

Surprisingly, the isolation rate of *E. coli* was significantly higher in faecal samples (84.8%) compared to liver samples (66.7%), suggesting that faecal contamination is a primary source of transmission. Comparable findings were observed by a previous study that reported a 66% recovery rate of *E. coli* from cloacal swabs (Akond et al. 2009), thus emphasizing the importance of faecal management in controlling bacterial spread within poultry farms. Because faecal samples harbour a higher bacterial load, they should be prioritized in future surveillance programmes for a better understanding of the transmission routes.

The present investigation reported a higher prevalence of *E. coli* infections in live birds (82.2%) compared to dead birds (67.5%), suggesting that live broilers actively shed the pathogen. This is consistent with another study (Yousef et al. 2023). These findings reinforce the need for better biosecurity and hygienic practices in poultry farms to minimize the spread of the pathogen from live birds to humans.

For over 50 years, antibiotics have been widely used in animal production for therapeutic purposes, disease prevention and growth promoters (Abreu et al. 2023). In this study, *E. coli* isolates showed a 100% resistance to TE, CLOX and COT, mirroring findings from previous studies conducted in Bangladesh (Sarker et al. 2019; Parvez et al. 2016; Amin et al. 2020; Ievy et al. 2020). Furthermore, E exhibited a high resistance rate at 91.8%, which is in agreement with similar studies (Al‐Salauddin et al. 2015; Rahman et al. 2020; Ievy et al. 2020). In contrast, CIP remained relatively effective, with a 74.1% susceptibility rate, whereas resistance to AMC and AZM was moderate at 43.52% and 25.88% respectively. These findings are consistent with two different studies conducted in Bangladesh (Sarker et al. 2019; Saha et al. 2020). Notably, no resistance was detected to GEN, a finding that aligns with a previous study (Ievy et al. 2020). This variability in resistance patterns suggests that although older antibiotics are losing efficacy, newer, less commonly used ones such as AZM and GEN may still offer viable alternatives for treatment. However, the gradual emergence of resistance even to these alternatives should serve as a call to action for more prudent antibiotic use.

The correlation and coexistence patterns observed among the tested antimicrobial agents in *E. coli* provide insights into the dynamics of AMR. The positive correlation, particularly between AMC with AZM and E, suggests potential co‐selection or cross‐resistance mechanisms. Such associations may arise from linked resistance genes on mobile genetic elements like plasmids, which facilitate co‐resistance to multiple antibiotics (Partridge et al. 2018). The negative correlation between CIP and GEN may indicate different evolutionary pathways or selective pressure‐driving resistance, as resistance to fluoroquinolones often involves specific mutations that do not overlap with aminoglycoside resistance (Jacoby 2005).

The coexistence network further emphasizes the frequent co‐occurrence of several antimicrobial agents, likely due to shared resistance determinants on conjugative plasmids (Gama et al. 2020). In contrast, GEN's isolated position suggests a less common association with other agents, possibly reflecting different usage patterns or resistance mechanisms. The probable implication is that antibiotics whose resistance has not coexisted with others might be useful in treatment.

Although molecular data were not collected in this study, the observed resistance patterns noted could be due to particular genetic mechanisms. These include the presence of β‐lactamase genes, colistin resistance genes or tetracycline resistance genes, which are frequently associated with mobile genetic elements like plasmids, integrons and transposons (Partridge et al. 2018; Wang et al. 2018). These elements are responsible for spreading MDR through horizontal gene transfer within and between bacterial populations (von Wintersdorff et al. 2016; Frost et al. 2005).

This study offers several key strengths. First of all, it provides up‐to‐date and region‐specific data on the prevalence and AMR of *E. coli* in broiler chickens in Sylhet, a major poultry‐producing hub in Bangladesh with limited prior surveillance. Secondly, by including a variety of sample types, such as cloacal swabs, faeces, intestines and liver tissues from both live and dead birds, the study enables a more comprehensive assessment of *E. coli* distribution across different anatomical sites. Finally, we incorporated the correlation and co‐resistance patterns, offering valuable insight into the possible co‐selection and transmission of resistance patterns. These findings contribute to a more informed understanding of AMR in the poultry sector and can support more targeted intervention strategies. This study supports both Bangladesh's National Action Plan on AMR and the WHO's Global Action Plan by providing localized resistance data from the animal health sector—key to the One Health approach. The findings highlight the public health risks posed by MDR *E. coli* in poultry, which may spread to humans via contaminated meat, the environment or direct contact.

Despite the valuable findings, one primary limitation of this study is the small number of samples, which may not fully represent the broader population of broiler chickens in the study area. Additionally, the study was restricted to a specific geographical area, which limits the generalizability of the findings to other regions where farming practices and biosecurity measures may differ. Moreover, although antimicrobial susceptibility testing provided valuable insights, only eight antibiotics were assessed, which may have constrained the ability to detect a broader range of resistance patterns. Furthermore, we did not discriminate between pathogenic and commensal *E. coli* strains (including those from faecal swabs), which limits interpretation of their public health significance. In addition, our study completely depended on phenotypic detection of AMR, which does not fully uncover the underlying genetic mechanisms. The inclusion of fingerprinting method or virulent gene profiling for comparing the pathogenic and commensal strains would offer valuable insights into strain‐level differences and pathogenic potential. The use of molecular approaches to identify specific AMR genes would strengthen our findings and provide valuable insights into the resistance determinants. Finally, the cross‐sectional design only provides a snapshot in time, underscoring the need for longitudinal studies to evaluate the trends in AMR over time.

## 5 Conclusion

This study reveals a high prevalence of antimicrobial resistance (AMR) *E. coli*, including multidrug‐resistant strains in broiler chickens from the Sylhet district of Bangladesh. Notably, the present investigation reported complete resistance to TE, CLOX and COT, raising concerns about the efficacy of these drugs for managing bacterial infections in poultry. Although CIP and GEN showed comparatively lower resistance rates, the widespread resistance overall underscores the urgent need for stricter regulation, veterinary oversight and farmer education on responsible antibiotic use. Continued AMR surveillance is essential to guide effective treatment and reduce the risk of zoonotic transmission. Future research should include molecular resistance profiling to uncover genetic mechanisms, broader antibiotic screening and longitudinal studies to better understand resistance mechanisms and inform targeted interventions.

## Author Contributions

**Md. Siddiqul Islam and Md. Anwar Hossain**: conceptualization, project administration, funding acquisition, supervision, resources and validation. **Manna Roy, Jahid Hasan Tipu, Sharmin Sultana Misty and Md Altafur Rahman**: methodology, investigation and original draft preparation. **Ahsan Raquib, Obaidul Islam, Raju Kurmi and Md Ashraful Islam**: software, data curation, formal analysis, data analysis and visualization. **All the authors**: writing – review and editing.

## Ethics Statement

Animal handling in this study was performed in accordance with the current legislation of Bangladesh (Cruelty to Animals Act 1920, Act No. I of 1920, Government of the People’s Republic of Bangladesh).The authors confirm that all efforts were made to ensure animal welfare and humane treatment.

## Conflicts of Interest

The authors declare no conflicts of interest.

## Peer Review

The peer review history for this article is available at https://www.webofscience.com/api/gateway/wos/peer‐review/10.1002/vms3.70576

## Supporting information




**Supporting Table 1**: Geographical coordinates (latitude and longitude) of the farms included in this study.
**Supporting Table 2**: Number of samples that tested positive in different groups in this study.
**Supporting Table 3**: List of antibiotic discs (Hi media, India) used in this study.
**Supporting Table 4**: Diameter of zone of inhibition interpretative standards provided by CLSI (2020).
**Supporting Table 5**: Antibiotic susceptibility patterns of 85 *E. coli* isolates from broiler chickens that were positive in PCR in the present study.
**Supporting Figure 1**: Isolation and identification of *E. coli* by culture, different biochemical tests, gramstain, and PCR methods in this study.
**Supporting Figure 2**: Results of antibiotic sensitivity test by disc diffusion method, where CIP−5 = ciprofloxacin (5 µg), AZM−30 = azithromycin (30 µg), GEN−10 = gentamycin (10 µg), E−15 = erythromycin (15 µg), TE−30 = tetracycline (30 µg), COX−1 = cloxacillin (1 µg), AMC−30 = amoxicillin/clavulanic acid (30µg), COT−25 = co−trimoxazole (25 µg).

## Data Availability

The data that support the findings of this study are available from the corresponding author (Md. Siddiqul Islam) upon reasonable request.
