# Supplementary material for: Prevalence and Antimicrobial Resistance Patterns of Escherichia coli Isolated From Broiler Chickens in Sylhet District of Bangladesh
Source: Vet Med Sci. 2025 Sep 8;11(5):e70576. doi: 10.1002/vms3.70576 (PMC12416310; doi:10.1002/vms3.70576)
Supplement: Supplementary file 1 — Supporting Table 1: Geographical coordinates (latitude and longitude) of the farms included in this study. Supporting Table 2: Number of samples that tested positive in different groups in this study. Supporting Table 3: List of antibiotic discs (Hi media, India) used in this study. Supporting Table 4: Diameter of zone of inhibition interpretative standards provided by CLSI (2020). Supporting Table 5: Antibiotic susceptibility patterns of 85 E. coli isolates from broiler chickens that were positive in PCR in the present study. Supporting Figure 1: Isolation and identification of E. coli by culture, different biochemical tests, gramstain, and PCR methods in this study. Supporting Figure 2: Results of antibiotic sensitivity test by disc diffusion method, where CIP−5 = ciprofloxacin (5 µg), AZM−30 = azithromycin (30 µg), GEN−10 = gentamycin (10 µg), E−15 = erythromycin (15 µg), TE−30 = tetracycline (30 µg), COX−1 = cloxacillin (1 µg), AMC−30 = amoxicillin/clavulanic acid (30µg), COT−25 = co−trimoxazole (25 µg). [file VMS3-11-e70576-s001.docx]

**Supplementary materials by Roy et al., 2024**

**Table S1:** Geographical coordinates (latitude and longitude) of the farms included in this study

| **Farm ID** | **Location** | **Latitude** | **Longitude** |
| --- | --- | --- | --- |
| Farm A | Beanibazar | 24°50'11.9"N | 92°09'46.6"E |
| Farm B | Zakiganj | 24°53'32.3"N | 92°27'31.3"E |
| Farm C | Gowainghat | 25°06'10.8"N | 91°58'25.4"E |
| Farm D | Jaintapur | 25°09'15.7"N | 92°07'47.0"E |
| Farm E | Kanaighat | 24°55'56.93"N | 92°11'14.05"E |

**Table S2:** Number of samples that tested positive in different groups in this study

| **Categories** | **Sample tested (n)** | **Sample tested positive (n)** |
| --- | --- | --- |
| **Farm (location)** |  |  |
| Farm A (Beanibazar) | 26 | 24 (92.3%) |
| Farm B (Zakiganj) | 33 | 26 (78.7%) |
| Farm C (Gowainghat) | 38 | 25 (65.8%) |
| Farm D (Jaintapur) | 15 | 12 (80.0%) |
| Farm E (Kanaighat) | 18 | 14 (77.7%) |
| **Bird status** |  |  |
| Live | 90 | 74 (82.2%) |
| Dead | 40 | 27 (67.5%) |
| **Sample type** |  |  |
| Cloacal swab | 44 | 35 (79.5%) |
| Feces | 46 | 39 (84.8%) |
| Liver | 21 | 14 (66.7%) |
| Intestine | 19 | 13 (68.4%) |

**Table S3:** List of antibiotic discs (Hi media, India) used in this study

| **Name of the antibiotic disc** | **Antibiotic group** | **Disc concentration** |
| --- | --- | --- |
| Ciprofloxacin (CIP) | Fluoroquinolone | 5 μg |
| Gentamicin (GEN) | Aminoglycoside | 10 μg |
| Erythromycin (E) | Macrolides | 15 μg |
| Azithromycin (AZM) | Macrolides | 30 μg |
| Tetracycline (TE) | Tetracyclines | 30 μg |
| Amoxycillin/Clavulanic acid (AMC) | Penicillin | 30 μg |
| Cloxacillin (COX) | Penicillin | 1 μg |
| Co-trimoxazole (COT) | Sulphonamides | 25 μg |

**Table S4:** Diameter of zone of inhibition interpretative standards provided by CLSI (2020)

| **Antibiotic**  **discs** | **Antibiotic group** | **Resistance** | **Intermediate** | **Sensitive** |
| --- | --- | --- | --- | --- |
| Ciprofloxacin | Fluoroquinolone | ≤15 mm | 16-20 mm | ≥21 mm |
| Gentamicin | Aminoglycoside | ≤12 mm | 13-17 mm | ≥18 mm |
| Erythromycin | Macrolides | ≤13 mm | 14-17 mm | ≥18 mm |
| Azithromycin | Macrolides | ≤12 mm | 15-17 mm | ≥13 mm |
| Tetracycline | Tetracyclines | ≤14 mm | 15-18 mm | ≥19 mm |
| Amoxycillin/Clavulanic acid | Penicillin | ≤19 mm | 20-22 mm | ≥23 mm |
| Cloxacillin | Penicillin | ≤17 mm | 18-24 mm | ≥25 mm |
| Co-trimoxazole | Sulphonamides | ≤11 mm | 11-13 mm | ≥14 mm |

**Table S5**: Antibiotic susceptibility patterns of 85 E. coli isolates from broiler chickens that were positive in PCR in the present study

| **Antimicrobial agents** | **Resistant,**  **n (%)** | **Intermediate,**  **n (%)** | **Sensitive,**  **n (%)** |
| --- | --- | --- | --- |
| Ciprofloxacin | 63 (74.1%) | 0 (0%) | 22 (25.9%) |
| Gentamicin | 0 (0%) | 59 (69.4%) | 26 (30.6%) |
| Erythromycin | 78 (91.8%) | 0 (0%) | 7 (8.2%) |
| Azithromycin | 22 (25.9%) | 13 (15.3%) | 50 (58.8%) |
| Tetracycline | 85 (100%) | 0 (0%) | 0 (0%) |
| Amoxycillin | 37 (43.5%) | 18 (21.2%) | 30 (35.3% |
| Cloxacillin | 85 (100%) | 0 (0%) | 0 (0%) |
| Co-trimoxazole | 85 (100%) | 0 (0%) | 0 (0%) |

| 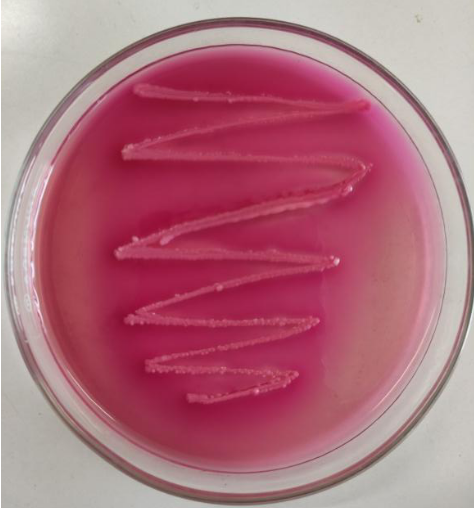 | 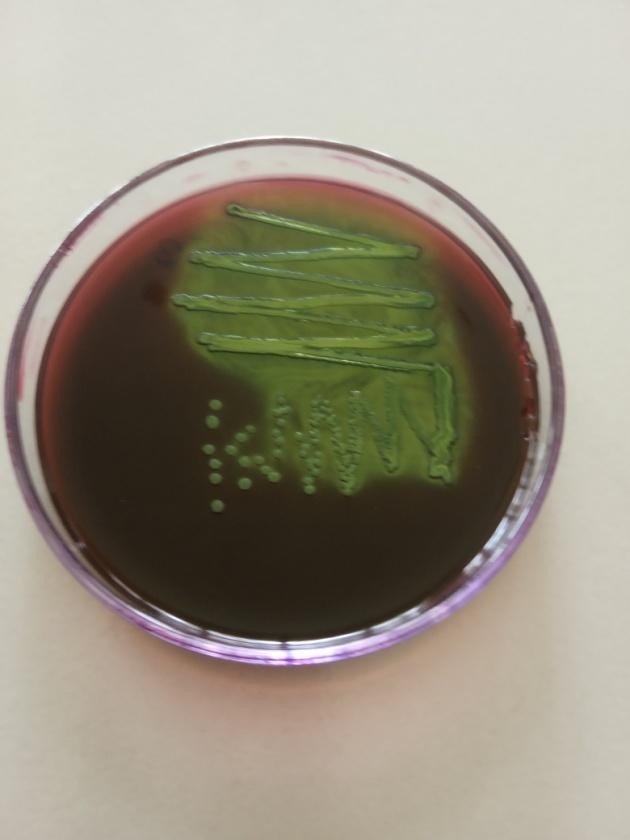 | |
| --- | --- | --- |
| 1. Colonies of *E. coli* on MacConkey agar. | 1. Colonies of *E. coli* on EMB agar media. | |
| 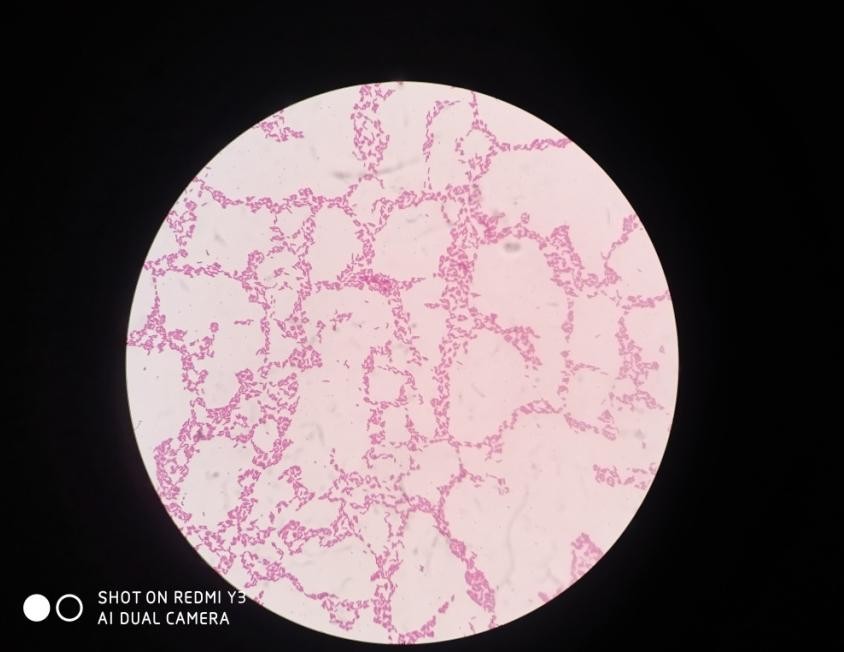 | 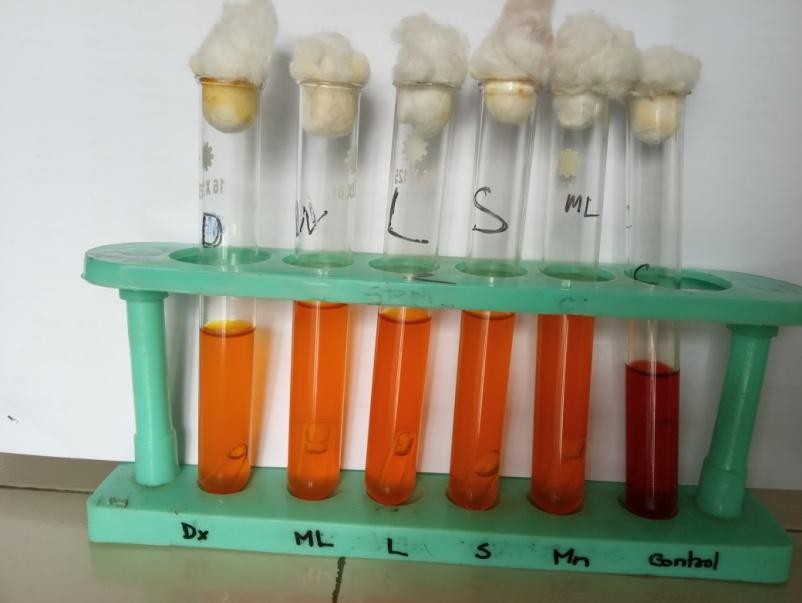 | |
| 1. Gram staining of *E. coli* under a light microscope (100X). | 1. Biochemical testing was conducted using five basic sugars: dextrose (Dx), maltose (ML), lactose (L), sucrose (S), and mannitol (Mn), with a control group. | |
| 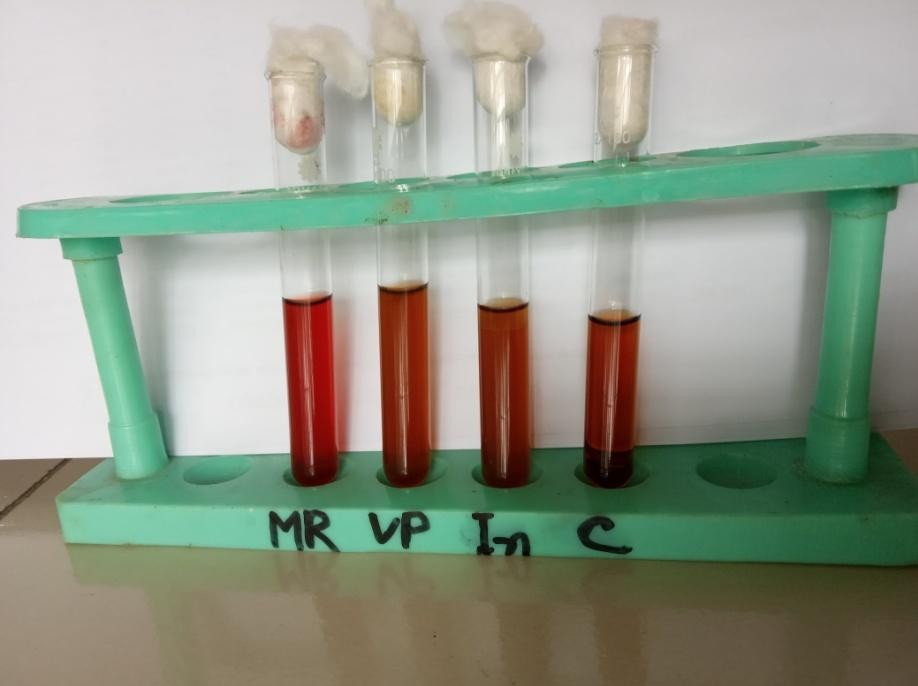 | **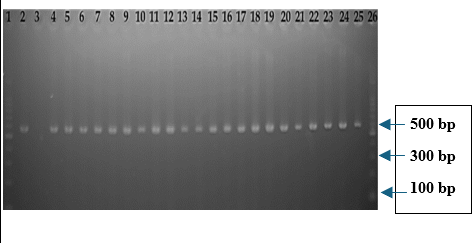** | |
| 1. *E. coli* identification was confirmed by biochemical tests: positive for indole, catalase, and methyl red, and negative for Voges-Proskauer. | (F) Gel documentation of amplified PCR products targeting the *malB* gene of *E. coli*. Lane 1 and 26: 1000 bp DNA ladder, Lane 2: positive control, Lane 3: negative control, Lane 4-25: amplified PCR products with expected band size of 585 bp. | |
| **Figure S1:** Isolation and identification of *E. coli* by culture, different biochemical tests, gram stain, and PCR methods in this study. | | |
| 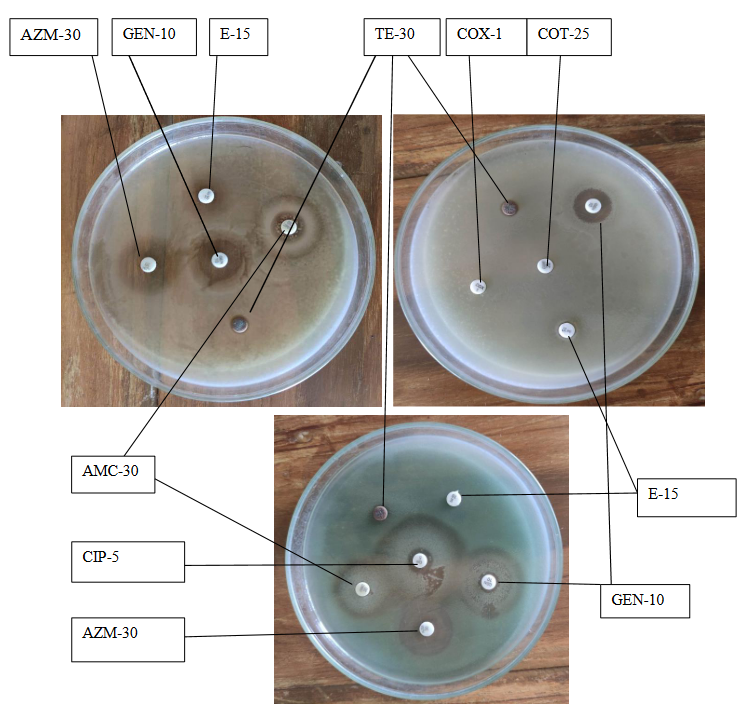 | |  |
| **Figure S2:** Results of antibiotic sensitivity test by disc diffusion method, where CIP-5= ciprofloxacin (5 µg), AZM-30= azithromycin (30 µg), GEN-10= gentamycin (10 µg), E-15= erythromycin (15 µg), TE-30= tetracycline (30 µg), COX-1= cloxacillin (1 µg), AMC-30= amoxicillin/clavulanic acid (30µg), COT-25=co-trimoxazole (25 µg). | |  |
